# Clinical Research on Neglected Tropical Diseases: Challenges and Solutions

**DOI:** 10.1371/journal.pntd.0004853

**Published:** 2016-11-03

**Authors:** Marleen Boelaert, The NIDIAG Consortium

**Affiliations:** Institute of Tropical Medicine, Antwerp, Belgium; Yale School of Public Health, UNITED STATES

Research pertaining to the neglected tropical diseases (NTDs) poses specific challenges that are linked to the diseases investigated, infrastructure (or lack thereof), culture, social-ecological systems, conflicting health policies, and ethics requirements, among others [1–3]. In 2010, the European research network on better diagnosis for neglected infectious diseases (NIDIAG consortium; see: http://www.nidiag.org) was launched to carry out collaborative research with an emphasis on NTDs. NIDIAG’s overarching goal was to generate evidence about the spectrum of causal pathogens of selected syndromes in different epidemiologic settings in order to improve the diagnosis and management.

The NIDIAG consortium is composed of research groups from African and Asian countries endemic for human African trypanosomiasis, schistosomiasis, soil-transmitted helminthiasis, visceral leishmaniasis (VL), and other NTDs, together with European biomedical and clinical researchers working on the same topics. The research is facilitated by a grant from the European Union (EU) within the Seventh Framework Programme (FP7). The consortium launched its work on November 1, 2010, for an initial five-year period and an additional six-month no-cost extension until April 30, 2016.

In the current issue of *PLoS Neglected Tropical Diseases*, a collection of articles [4–8, 39]—mainly prepared in the style of symposia—is published, in which we would like to share experiences and lessons from the NIDIAG consortium that range from designing, setting up, implementing, and evaluating multiple clinical studies in partner countries in the global South. In this editorial, we provide an umbrella, discuss perceived challenges in the field of NTD research, and propose solutions on how these issues were overcome during the conduct of the NIDIAG studies. We hope that this article collection will stimulate others working on clinical-diagnostic issues of NTDs to further improve patient management and population-based control and elimination.

## Challenge #1: Binary Thinking and Syndromic Management of NTDs

NTDs have often been discussed from an exclusive pathogen or “single disease” perspective rather than considering the potential etiologies of specific clinical syndromes with which a patient presents to a health care center. Additionally, a distinction was made between those NTDs that are thought to be controllable at the population level by means of mass drug administration (e.g., five to six rounds of albendazole plus ivermectin administered to all community members aged five years and above against lymphatic filariasis) and preventive chemotherapy (e.g., annual treatment of school-aged children with praziquantel against schistosomiasis) on one hand, and, on the other hand, those NTDs that involve individual case finding and management (e.g., Buruli ulcer) [9]. Recently, increasing attention has been given to the issues of co-endemicity, coinfection, and comorbidity [10–12], but generally, binary thinking prevails with regard to the identification, management, control, and elimination of NTDs. It should be noted, however, that for the patients suffering from those problems, the specific causal pathogens are of lesser importance. It is the backache, headache, and persistent diarrhea that prevent rural dwellers from working their land, the threat to lose their children to a fatal febrile illness, and the intractable scar in a young daughter’s face that may isolate her socially for the remainder of her life. These are some of the genuine problems of NTDs from the patient’s perspective.

Suppose your child has been feverish for more than two weeks, but you are sent back home from the primary health care center without a treatment because the rapid diagnostic tests (RDTs) for malaria and VL done at the center were negative: that is profoundly unsatisfactory for any parent or caregiver. This issue is far from being resolved. The full extent and the changing patterns of the “non-malarial fever” have been acknowledged by the global health community only recently [13,14]. Although fevers due to causes other than malaria have been investigated by researchers for decades [15,16], the actual, true burden has not been fully appreciated until the introduction of highly sensitive RDTs and their broad application even in remote health care centers in endemic settings [17]. Indeed, malaria is now ruled out in a large number of patients with febrile syndromes in tropical regions, and, immediately, a large spectrum of possible differential diagnoses unfolds [15,18–20]. However, even in the case of laboratory-confirmed detection of a pathogen in a patient specimen, recent research has brought to light that the attribution of causation remains daunting [21,22].

**Fig 1:**
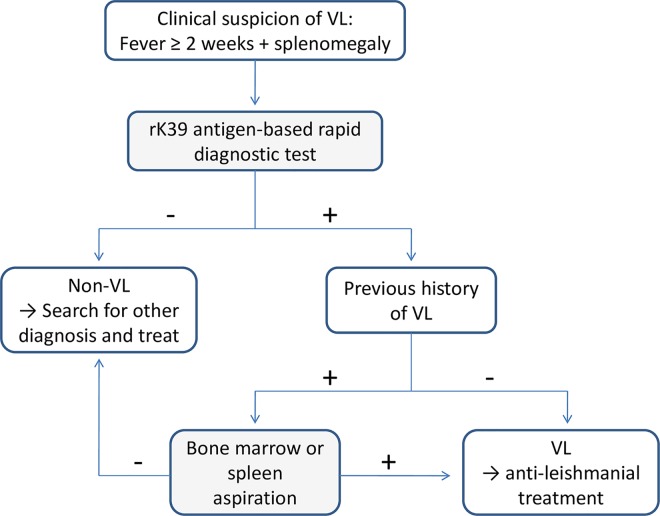
Binary algorithm for the diagnostic evaluation of visceral leishmaniasis (VL, also known as kala-azar) in South Asia. Investigations for alternative pathogens that may give rise to a similar clinical syndrome are not considered in such an algorithm.

Unfortunately, the laboratories in resource-constrained settings are often not able to give guidance for therapeutic management. In practice, patients usually receive a cocktail of drugs, including antibiotics, on an empiric basis, and they are told to come back if they do not get better [17]. Syndromic management is probably a better approach in such cases, as has been shown for sexually transmitted infections [23] and integrated management of childhood illness [24]. However, it has also been demonstrated that these syndromic guidelines are frequently not being followed in endemic areas, in particular if the health care personnel do not receive specific training pertaining to these guidelines and are not involved in the process of developing such guidance tools [25]. Thus far, the evidence base for a syndromic approach toward common clinical syndromes in areas characterized by a high prevalence of specific NTDs is flimsy at best. It is important to note that clinical syndromes are defined by a frequently encountered combination of clinical signs and symptoms, such as long-lasting fever, abdominal complaints, or respiratory signs suggestive of infection, and not by a specific etiology. Hence, a “syndromic approach to NTDs” pertains to clinical syndromes that occur frequently in tropical and subtropical areas and where it may be anticipated that a large proportion of these diseases are caused by NTDs. Indeed, when a patient with a given clinical symptomatology presents to a rural health care center, binary diagnosis-treatment strategies are commonly employed, which frequently focus on ruling in or out a specific disease, whereas other causes of a similar symptomatology are not sufficiently considered in existing algorithms. An example of such a strategy is shown in Fig 1, i.e., a recommended algorithm for the diagnosis of VL (also known as kala-azar) in endemic areas. Such inflexible “yes/no algorithms” are often of limited value to clinicians working in settings where multiple NTDs and other infections coexist. Hence, the NIDIAG consortium set out to employ a syndromic approach to elucidate the differential diagnosis of three NTD-related syndromes, namely (i) persistent fever [26,27]; (ii) neurological disorders [28]; and (iii) persistent digestive disorders [29,30].

## Challenge #2: Implementing Research Compliant with Good Clinical Practice (GCP) and Good Clinical Laboratory Practice (GCLP)

The idea for the syndrome-specific NIDIAG investigations was straightforward: design and implement a series of prospective clinical studies, recruit patients with one of the three syndromes at primary care level in NTD-endemic areas, and try to reach a final diagnosis in the greatest possible number, if necessary, through the shipment of samples to reference laboratories in-country or abroad. The knowledge on specific pathogens causing the pre-specified syndrome could then be used in a second stage to elaborate clinical-diagnostic algorithms and to provide guidance for a given epidemiologic setting.

Yet, a seemingly straightforward idea can become complex, particularly when the ambition is to employ a syndromic approach, to work in multiple study sites, to be compliant with the essential ethical principles of respect for persons, beneficence, and justice [31], and to adhere to good clinical practice (GCP) and good clinical laboratory practice (GCLP) standards throughout. Needless to say that the challenges are plentiful; that they are of scientific, ethical, legal, regulatory, and logistic nature; and that they are complex in today’s scenario of international research [32]. Indeed, clinical research conducted in resource-constrained settings has to come to grips with essential challenges related to community engagement, participants’ vulnerability, upgrade of local clinical and laboratory infrastructures, data sharing, sample storage and shipment abroad, capacity transfer, and benefit sharing. For instance, a recent systematic review has underscored that there is considerable heterogeneity across sub-Saharan Africa regarding biobanking issues, with often conflicting regulations and guidelines in different countries, which are likely to negatively affect international collaborative efforts [33]. Additionally, the harmonization of diagnostic and clinical procedures in multi-country studies is frequently not sufficiently addressed and, particularly in low- and middle-income countries, training and adherence to GCP/GCLP guidelines is particularly challenging [34].

With regard to diagnostic accuracy, core requirements are the establishment of a quality assurance system that includes internal and external quality control and monitoring activities [35]. This is an important prerequisite to avoid misleading research results, as has recently been underscored by a nationwide study from the Democratic Republic of the Congo, where more than 60% of laboratories failed to reach an acceptable score for the microscopic diagnosis of malaria and human African trypanosomiasis [36]. The study also demonstrated better results at sites where regular training and external quality assessments were carried out.

Hence, a legacy of the NIDIAG consortium is the efforts made with harmonizing study protocols and the development and validation of a large number of standard operating procedures (SOPs) that were employed in the context of this project. Indeed, 80 specific SOPs were developed by the NIDIAG consortium, mainly in the domains of laboratory (*n* = 59), data management, documentation, quality control (*n* = 13), and clinical aspects (*n* = 8). A summary of all the SOPs, stratified by syndrome, is given below (Table 1). As can be seen, many SOPs were used across syndromes, which demonstrates harmonization of quality systems beyond specific boundaries. Other SOPs are disease- and syndrome-specific, which is to be expected, as the diagnosis, management, and treatment are idiosyncratic. We believe that sharing these SOPs on an open-access platform and providing additional specific information in a set of symposia articles in the current issue of *PLoS Neglected Tropical Diseases* [4–8] will contribute to facilitate and further strengthen GCP/GCLP-compliant clinical research by colleagues working in this field. Here, we share experiences and lessons from the NIDIAG consortium that range from designing, setting up, implementing, and evaluating multiple GCP/GCLP-compliant clinical studies in low- and middle-income countries in Africa and Asia. Hence, it is our hope that the various SOPs will be used, adapted to specific local contexts, and further improved in the interest of the most neglected patients—those living in NTD-affected areas.

**Table 1:** Overview of SOPs for the three syndromes addressed in the NIDIAG consortium.

Type of SOP	Purpose of SOP	Intended user	Use in NIDIAG syndromes		
			Persistent fever	Neurological disorders	Persistent digestive disorders
Clinical	Assessing inclusion / exclusion criteria	Site investigators	√	√	√
Clinical	History taking	Site investigators	–	–	√
Clinical	Clinical examination (at baseline and during follow-up)	Site investigators	√	√	√
Clinical	Selection of controls	Site investigators	–	–	√
Clinical	Clinical management of patients	Site investigators	–	√	√
Clinical	Patient recruitment and patient flow	Site investigators	–	–	√
Clinical	Performing lumbar puncture	Site investigators	√	√	
Clinical	Performing bone marrow aspirate	Site investigators	√	–	–
Laboratory	Diagnostic sample flow	All research staff	√	√	√
Laboratory	Urine sampling	Study nurses	√	√	√
Laboratory	Blood sampling (blood cultures, serum, heparin, and EDTA whole blood)	Study nurses	√	√	–
Laboratory	How to obtain a stool sample	Site investigator and study nurses	–	–	√
Laboratory	Lymph node aspiration	Study nurses	√	√	–
Laboratory	Hemoglobin measurement with HemoCue Hb 301	Laboratory technicians	√	√	–
Laboratory	White blood cell count and differentiation with HemoCue WBC DIFF	Laboratory technicians	√	√	–
Laboratory	Thick blood film, Giemsa staining, and microscopic examination	Laboratory technicians	√	√	–
Laboratory	White blood cell count in CSF with counting chamber "Uriglass" (Menarini)	Laboratory technicians	–	√	–
Laboratory	Gram staining of CSF samples	Laboratory technicians	–	√	–
Laboratory	Leukocyte differentiation on a Giemsa-stained CSF smear	Laboratory technicians	–	√	–
Laboratory	Use of the Reflotron Plus (Roche) biochemical analyzer	Laboratory technicians	√	√	–
Laboratory	Glucose test (glycaemia)—HumaSens glucometer	Laboratory technicians	–	√	–
Laboratory	CSF culture in trans-isolate (T-I) medium	Laboratory technicians	–	√	–
Laboratory	CSF culture in pediatric Bact/Alert bottles	Laboratory technicians	–	√	–
Laboratory	CD4+ cell count (Dynal T4 Quant Kit/Invitrogen)	Laboratory technicians	–	√	–
Laboratory	Storage of bacterial isolates	Laboratory technicians	√	–	–
Laboratory	Use of urine test strips «Multistix 10 SG» (Siemens Healthcare Diagnostics)	Laboratory technicians	√	–	–
Laboratory	Direct fecal smear technique	Laboratory technicians	–	–	√
Laboratory	Preparation of aliquots for molecular testing	Laboratory technicians	–	–	√
Laboratory	Fresh trypanosome examination in lymph node fluid	Laboratory technicians	–	√	–
Laboratory	Mini anion exchange centrifugation technique (mAECT)	Laboratory technicians	√	√	–
Laboratory	Modified single centrifugation	Laboratory technicians	√	√	–
Laboratory	Capillary tube centrifugation technique	Laboratory technicians	√	√	–
Laboratory (index test)	Card agglutination test for *Trypanosoma brucei gambiense* (CATT/*T*.*b*. *gambiense*)	Laboratory technicians	√	√	–
Laboratory (index test)	Performing the SD Bioline HAT test (Standard Diagnostics)	Laboratory technicians	√	√	–
Laboratory (index test)	Performing the Gambiense HAT Sero-K-Set test (Coris BioConcept)	Laboratory technicians	√	√	–
Laboratory	CareStart Malaria pLDH (Access Bio)	Laboratory technicians	√	√	–
Laboratory	Malaria SD BIOLINE Ag Pf/Pan (SD 05FK60)	Laboratory technicians	√	√	–
Laboratory	Performing the DAT	Laboratory technicians	√	–	–
Laboratory	Inoculating and growing cultures of *Leishmania donovani* from body fluids and tissue aspirates	Laboratory technicians	√	–	–
Laboratory	Detecting *L*. *donovani* in body fluids and tissue aspirates	Laboratory technicians	√	–	–
Laboratory (index test)	Performing the rK28 from EASE-Medtrend	Laboratory technicians	√	–	–
Laboratory (index test)	Performing the rK39 IT LEISH (Bio-Rad)	Laboratory technicians	√	–	–
Laboratory (index test)	Performing the Salmonella Typhi IgM/IgG (SD Bioline)	Laboratory technicians	√	–	–
Laboratory (index test)	Performing the Typhidot Rapid IgM (Reszon Diagnostics)	Laboratory technicians	√	–	–
Laboratory (index test)	Performing the Test-it Typhoid IgM (Life Assay)	Laboratory technicians	√	–	–
Laboratory (index test)	Performing the *Leptospira* IgG/IgM (SD Bioline)	Laboratory technicians	√	–	–
Laboratory (index test)	Performing the Test-it Leptospirosis IgM (Life Assay)	Laboratory technicians	√	–	–
Laboratory	Syphilis–RPR test on Macro-Vue cards	Laboratory technicians	–	√	–
Laboratory (index test)	SD Bioline Syphilis 3.0 (Standard Diagnostics)	Laboratory technicians	–	√	–
Laboratory	India ink staining for *Cryptococcus* in CSF	Laboratory technicians	–	√	–
Laboratory	*Cryptococcus* antigen latex agglutination test	Laboratory technicians	–	√	–
Laboratory (index test)	*Cryptococcus* Antigen (CrAg) Lateral Flow Assay (Immunomycologics)	Laboratory technicians	–	√	–
Laboratory	Determine HIV-1/2	Laboratory technicians	√	√	–
Laboratory	Uni-Gold HIV	Laboratory technicians	–	√	–
Laboratory	DoubleCheckGold HIV 1&2	Laboratory technicians	–	√	–
Laboratory	CSF culture for mycobacteria in BBL MGIT medium (BD) supplemented with OADC and PANTA	Laboratory technicians	–	√	–
Laboratory	Hot Ziehl-Neelsen staining for detection of mycobacteria in CSF	Laboratory technicians	–	√	–
Laboratory	Sputum collection, smear and hot Ziehl-Neelsen staining for detection of mycobacteria	Laboratory technicians	√	√	–
Laboratory	Kato-Katz technique	Laboratory technicians	–	–	√
Laboratory	Mini-FLOTAC technique	Laboratory technicians	–	–	√
Laboratory	Formalin-ether concentration technique	Laboratory technicians	–	–	√
Laboratory	Kinyoun staining technique	Laboratory technicians	–	–	√
Laboratory	Modified acid-fast staining technique	Laboratory technicians	–	–	√
Laboratory	Koga agar plate culture	Laboratory technicians	–	–	√
Laboratory	Baermann technique	Laboratory technicians	–	–	√
Laboratory (index test)	Crypto-Giardia Duo-Strip (Coris BioConcept) rapid diagnostic test	Laboratory technicians	–	–	√
Laboratory (index test)	Point-of-care circulating cathodic antigen (POC-CCA) urine cassette test for the diagnosis of *Schistosoma mansoni*	Laboratory technicians	–	–	√
Document	Obtaining informed consent	Site investigators	√	√	√
Document	Patients and samples numbering system in NIDIAG studies	All research staff	√	√	√
Document	Management of study documents	All research staff	√	√	√
Quality	SOP on SOP	SOP authors and reviewers	√	√	√
Quality	Internal quality control activities	All research staff	√	√	√
Quality	External monitoring	All research staff	√	√	√
Quality	Laboratory monitoring	Site investigators; laboratory technicians; monitors	√	√	√
Quality	Min/max thermometer	Laboratory technicians	√	√	√
Quality	Stock management	Laboratory technicians	√	√	√
Quality	Handling of expired and disqualified products	Laboratory technicians	√	√	√
Quality	Handling and storage of RDTs	Laboratory technicians	√	√	√
Data	Completing case report forms (CRFs)	Site investigators	√	√	–
Data	Data management	Data entry clerks	√	–	√

Ag: antigen; CRF: case report form; CSF: cerebrospinal fluid; DAT: direct agglutination test; EDTA: ethylenediaminetetraacetic acid; HAT: human African trypanosomiasis; Ig: immunoglobin; IT: immunochromatographic test; LDH: lactate dehydrogenase; RDT: rapid diagnostic test; SOP: standard operating procedure; RPR: rapid plasma reagin

## Challenge #3: Maintaining Improved Patient Care after Clinical Studies Have Come to an End

Not only the scientific findings of a clinical study, but also the study procedures and the learning process during the conduct of the study can contribute to improved patient care afterwards. SOPs that are developed in the first place to define and harmonize procedures (across operators, sites, and countries) within a specific clinical study could also be beneficial after the end of that study, because they might be used as practical training tools, and they might help achieving sustained improvement of clinical and diagnostic procedures. Nevertheless, there are many barriers for the consistent use of SOPs. During the course of a study, these include the fear that clinical autonomy will be reduced, underestimating the importance of harmonization of clinical and laboratory procedures, the fear that written guidelines will cause additional paperwork, lack of a culture of quality assurance systems, and lack of motivation to change practice [37]. After the end of a clinical study, and particularly in resource-constrained settings, there is a risk that quality improvements achieved during the study will erode when staff support, incentives, means, and monitoring suddenly come to an end [38].

Ownership and local leadership is paramount for sustained adherence to a quality system. Within the NIDIAG project, we have tried to contribute to the long-term sustainability of the quality system in the following ways: by doing the diagnostic testing as much as possible in the study sites; by investing in the training and remote support of local staff; by jointly developing SOPs that are readily adapted to specific contexts thanks to the input of local staff; and by transferring equipment to the study sites at the end of the study and ensuring sustained assistance for its maintenance. Although in the beginning, there was quite some skepticism from some staff regarding the introduction of a quality system based on GCP/GCLP and SOPs, the mood was very different at the end of the NIDIAG project. In the final consortium meeting done in March 2016 in Indonesia, several researchers spontaneously mentioned that getting to know GCP/GCLP in practice and engaging in a dialogue with study monitors and laboratory supervisors had been an enriching experience and assisted in building up local research capacity, which will facilitate further research and improved clinical-diagnostic management of NTDs in the global South.

## Conclusion

By sharing our experiences and lessons with GCP/GCLP-compliant clinical research in NTD-endemic settings and providing open access to a host of SOPs jointly developed during the NIDIAG project, we hope to help other researchers to overcome some of the specific challenges related to NTD research. In turn, we are confident that this will contribute to a culture of quality clinical research in NTD-endemic countries.
